# Extra Supplement—The Nursing Mirror

**Published:** 1890-05-24

**Authors:** 


### "The Hospital" Nursing Mirror.

Being the Extra Nursing Supplement of "The Hospital" Newspaper.

All Contributions for this Supplement should be addressed to the Editor, The Hospital, 140, Strand, London, W.C., and should have the word "Nursing" plainly written in left-hand top corner of the envelope.

### En Passant.

QUEEN CHARLOTTE'S HOSPITAL.—We constantly have queries about training for midwives and monthly nurses, so we are glad to extract the following from the 38th annual report of this well-known hospital: "The work of the school continues to progress in a satisfactory manner. Since its establishment no less than 2,370 persons have passed through a course of instruction, while last year a greater number of pupils entered than in any previous year. Sixty-eight medical pupils attended the hospital practice and received certificates required by the various examining bodies; 41 midwives were trained, and having passed examinations, were awarded certificates of proficiency; 149 monthly nurses were trained, and being found competent, received certificates. Of the above-mentioned midwives 18 passed the examination of the Obstetrical Society of London last year." The fee for midwives for 13 weeks is £26 5s.; the fee for monthly nurses is 11 guineas for eight weeks, or 15 guineas for 12 weeks. More than 18 out of 41 midwives ought to have passed the Obstetrical Society's examination.

ONE OF THE ÉLITE.—At a recent meeting of the British Nurses' Association it was announced that 800 nurses had applied for registration, and that these were mostly hospital matrons, sisters, and nurses, in fact, as one of the honorary secretaries of the Association put it, "the *élite* of the nursing profession." We have often given our opinion as to the quality of nurses likely to go on this register, and an important incident with regard to the above phrase has come to our knowledge. We keep at our office, for our private information, a "black list" of those nurses to whose discredit we have knowledge of certain facts. In January last a hospital matron wrote to us giving certain particulars with regard to a Miss Gertrude Johnstone, who had decamped from Teignmouth Infirmary, leaving the committee considerably in debt. In our issue for February 8th we warned all hospital authorities against this Miss Johnstone, and at the same time we looked her up in the British Nurses' Association list, and there the name appeared. A fortnight ago further information with regard to Miss Johnstone reached us, and we went to Guy's Hospital with which she said she was connected, but where we found she was utterly unknown. Then curiosity and that happy phrase about the *élite* tempted us to call at the British Nurses' Association office, and try and find out whether they had any idea where Miss Johnstone was trained (if ever!), or whether they simply never inquired into the references given by would-be members. Ignorance reigned supreme at the British Nurses' Association office; the clerk, who was the only person forthcoming, knew nothing. Then a letter to the Secretary elicited the fact that Miss Gertrude Johnstone *was* among the 800 *élite* who had applied for registration, and the Secretary requested further information about her. But we do not consider it our duty to make inquiries for the B.N.A. If they cannot even discover such a noted case as this Miss Johnstone's, which appeared both in the local and the London press, we really cannot undertake their work for them; we do not grudge them their *élite*, nor do we wish a single name struck off their register. And it appears useless for any poor creature once on the register to try and turn and fly. A well-known matron, who was on the General Council, sent in her resignation, and received a letter in reply stating that though always glad to add names, they never removed them! We leave the above facts to speak for themselves.

SHORT ITEMS.—An interesting meeting took place last Saturday at Lady George Hamilton's to discuss a proposed Home of Rest for Nurses. The subject is an important one, and is referred to elsewhere.—A meeting in aid of the Hammersmith District Nursing Association was held at Beavor Lodge on Tuesday, when Mrs. Garrett Anderson, M.D., and others spoke.—Miss Jessie Murray will open a Nursing Institute at Huddersfield in June.—Miss Arabella Stone has been appointed superintendent of the New Westminster Nursing Committee.

RURAL NURSING ASSOCIATION.—Sir Hy. Longley presided at a drawing-room meeting held at 11, Downing Street, last week, at the invitation of Lady Lucy Hicks-Beach and the Hon. Mrs. John Dundas, in favour of providing good trained nurses for the sick poor in rural districts. The speakers included the Earl of Yarborough, Lady Victoria Lambton, Mrs. Malleson, Dr. Lowe, Miss Broadbent, and others. The audience were numerous and fashionable; it is to be hoped that they gave largely to this very excellent charity. Mrs. Malleson is to be congratulated on the energy with which she is carrying forward this work.

THE METROPOLITAN ASSOCIATION.—The annual meeting of the Metropolitan and National District Nursing Association was held at Grosvenor House on the 9th inst., the Duke of Westminster in the chair. We were much struck by the fact that Miss Mansel, of Bloomsbury Square, was not mentioned—a stupid oversight on the part of some of the committee, who ought to see that thanks are given to all to whom they are due. Mrs. Dacre Craven and Miss Perse were praised, but Miss Mansel, who has worked at the central home for ten years, was passed over. It was, of course, a mere mistake, for Miss Mansel has just been chosen to train the Jubilee nurses. The report read was an excellent one; the Association is now free from debt, and the work is extending into many new districts. There are now fourteen smaller homes in connection with the central one, and last year nearly 20,000 visits were paid by the nurses. A munificent donation of £2,000 has been received from Mr. Henry Tate for the special purpose of founding a pension fund for nurses. The money has been invested in the names of the three trustees of the association, and the interest will be applied, as far as it goes, towards paying a portion of the annual premium for securing pensions, in connection with the National Pension Fund, for such nurses of the Association and its branches as may desire to avail themselves of its advantages.

ARRANGEMENTS FOR JUNE.—Correspondents please find in this paragraph the answers to their many queries about the presenting of certificates and the Junius S. Morgan Memorial. Either indoor or outdoor uniform can be worn at Marlborough House, but each nurse must arrive ready to be presented; there will be no cloak-room provided, and directly the nurse passes within the gates she must be ready to be received by the Princess. Therefore, those nurses who appear in indoor uniform must leave their bonnets, &c., at home, or at some station, and drive to Marlborough House. We believe the plan to be followed at two or three large London hospitals is to send the nurses down in an omnibus. The First Thousand are working well for the Memorial, and we acknowledge with thanks the sum of £1 from Miss S. A. Yorke towards this benevolent fund. The spirit displayed by the First Thousand is just what we should have expected from all that is best in the nursing world, for it shows benevolence, self-help, and thrift. One nurse asks if any purses will be provided; certainly, if any nurses wish it, but would it not be an excellent plan for each nurse to make the purse she presents? The purses can be any size or shape, and the money will have to be sent to the Pension Fund office, 8, King Street, Cheapside, E.C., from whence a receipt will be returned; it is this receipt, representing the amount collected, which must be put in the purse and presented to the Princess.

### Nurse Joyce.

### Part I.

"Take me home; oh, take me home!" Over and over the words were wailed out as the days dragged slowly by, and the hearts of the listeners ached.

In the little quiet room, at the top of the great hospital, far away from all sounds of life, lay Nurse Joyce, while over her was fought a hard combat with grim death. There were those in that hospital who would have counted the world immeasurably the poorer for her slipping out of it, and, therefore, put their shoulders manfully to the wheel to save her. It goes without saying that it was in the performance of her duty that Nurse Joyce had been stricken by—as was dreaded—a mortal wound.

We others have grown accustomed to the fact of those brave women who dauntlessly face dangers—in the shape of fell diseases—as deadly as are met by the soldier on the field of battle. We do not fully realise the splendidly-enduring courage of these sisters of ours. Some women there are, doubtless, who—in a sudden impulse of excitement—can perform an act of heroism (and be prostrated afterwards by the hysterical reaction); but it is the few—not the many—to whom is given the long suffering and the staying powers of endurance, which go to constitute that woman-warrior, the nurse—whom may God bless, prays many a one.

"There's news from London, Anne! Bad news, my dear," and the Rev. Oliver Joyce sat, with his hand shielding his face, reading an open letter at the parsonage breakfast-table.

"What is it, Oliver? Speak! I thought there'd be bad news, for I dreamed about silver last night!" and the parson's sister, a thin, precise young woman, waited, holding the tea-pot in mid-air.

"Lily has fallen ill at the hospital, desperately ill. Blood poisoning, they say, and there are grave fears."

"I knew it! I always knew it would happen!" cried Anne, who was one of those beings gifted with a marvellous omniscience, invariably kept locked in their own bosoms, until the world is as wise as themselves, then out it jumps. "Lily has brought it upon herself; if she lose her life there will be but herself to blame. No woman who rushes out into the world, deserting home duties, can expect but to encounter awful risks. Indeed, I'm always expecting to hear Lily has taken the small-pox, at least." Then Anne finished pouring out the Rev. Oliver's tea, and handed it to him.

Meals and their methodical serving were a species of religion with the elder Miss Joyce. She would hardly have permitted earthquakes to have interfered with the hour, and the accustomed appointments of the parsonage daily dinner. Her brother, of a less prosaic nature, sat with untasted breakfast, reading again and again the letter from the lady superintendent of the hospital where Nurse Joyce lay.

The three Joyces had been born and brought up in that same old stone-built parsonage among the Cumberland fells, where their father, the incumbent, had lived for years after his son Oliver succeeded him in the living, and then he passed away, fairly tired out by his long earthly journey. It was not until after his death that some faint ripples of the far-away great world's influences stirred in the heart of Lily, the youngest, a desire to be "up and doing." She rebelled hotly against the idea of being a human vegetable—like the green things about the place, that raised their heads out of the earth, just to come to their limit of perfection, and then to fade away back to that earth.

"A woman is made for home," said Anne, didactically, "and home is made for the woman. And there is the parish work, Lily; it should be enough for you. For me, I've the housekeeping. Oliver will never marry—he isn't cut out for that sort of thing." Neither, perhaps, was Anne herself. "It's quite clear that your place and mine are at home here;" but Lily could not see it.

"There are two of us, Anne, doing one woman's work. What's the work of this parish? Those outlying farms want none of us; it's a man's business—Oliver's—to visit them. And the handful of villagers cannot, and never will, suffice for the energy that is pent up in me. Besides, what has the desire been put in my heart for, and why have I the strength given me to perform it?" for Lily, straight and tall as her flower namesake, was also like it in the vigour and strength of her physique. It is an out-of-date mistake to talk about the lily's fragility; its slenderness is a poetical fiction. Examine the bold, sweeping, firm lines of stem and flower, and the vigour of the parent stalk, in the *arum* nearest to you. In it you will find no limp, unhealthy languishing. It is the same in the human lily—given a sound mind in a sound body. And the girl in question had both. The out-door life of the hills had filled her veins with blood of purest quality, from whence resulted the unflagging bodily strength which craved an outlet. But when Lily put her desire into words, and held firmly to it, that she would go to London and enter the nursing profession, Anne's disgust and obstinate opposition knew no bounds.

"It is a horrible idea!" declared the elder sister—one of those women who made a special point of fainting away at the mere sight of blood. "You little know what you'll have to go through"; and she shuddered, her pale, uniform complexion growing stone colour.

"Perhaps not," said Lily impetuously, the hot colour flaming into her bonny, sweet face; "but, so long as I can help some others to pull through their troubles, that should give me strength to face anything. Anne, you and I are as night and day; we can never agree, but don't let us quarrel. Why should we? Let me go my way; I pray you not to throw stumbling-blocks in that way, for I'm determined to walk it to the end, God helping me. Don't let my failure lie at your door."

"Girls!" Oliver Joyce turned round in the arm-chair, beside the window, from which the book-lover gazed out, now and then, at the misty hills, and listened to the burn singing jubilantly over the stones, at the end of the parsonage garden. It was an effort for the dreamy student to drag his thoughts from his books—of which Nature was the best loved—to enter into the subject his sisters were discussing, but a dim sense of duty to them constrained him. "Girls!" repeated he, "I've been considering Lily's project."

"Yes?" Anne bent eagerly forward, "and I'm sure, Oliver, you disapprove——"

"On the contrary, Anne, I should not presume to thwart a fellow-creature in such a solemn matter. If a voice has spoken in Lily's heart directing her to go out into the world, after the manner of the twelve disciples, whom our Lord sent forth to preach and to 'heal the sick,' then, let not us commit the sin of putting obstacles in her way." Oliver spoke with gravity, and Lily glanced gratefully at him, while Anne, a burning spot on either pale cheek, kept silence. It was but seldom Oliver interposed between the sisters, but when he did, she knew his decree was absolute.

Thus, it came to pass that Lily Joyce went forth in the springtime of her womanly beauty, and in the hey-day of her strength, to "do what she could," because the love of Christ constrained her. Will-power means success, and of that Lily had plenty. She rapidly made her way up through the drudgery pertaining to every profession, every trade; her skill and sympathetic gentleness becoming a by-word, and Lily was happier a thousand times than her sister, the precise Anne, who

> ". . . . . amid her narrow days,
> Moving about the household ways,
> In that dark house where she was born,"

hugged to herself the comfortable conviction that she was fulfilling her vocation in a womanly fashion, while Lily—well, Lily was carrying out her mission in a manner the reverse of feminine.

But there came a day when a bar was thrown across her career, and nurse Joyce fell out of the ranks; she was bloodpoisoned. Tired out by an unusual pressure of over-work, she who was ever indefatigable had contracted the subtle poison into her own system, which, enfeebled by fatigue, readily imbibed it.

*(To be continued.)*

### The Nurses' Bookshelf.

### Antiseptics for Nurses.*

This is an excellent little handbook, being an advance on others on the same subject, in so far that it is supplied with illustration, and examination questions at the end. The illustrations refer to the microscopical branch of the subject, one which only nurses of advanced education take up; but the questions at the end will be most useful to the lecturer who wishes to test the attention paid to his teaching. Mr. Stanmore Bishop writes well and brightly, and never allows his subject to become monotonous or dull; the book is exhaustive also, and deals with every needful point clearly yet fully. There is only one objection we have to bring forward, and that is whether it was necessary, so soon after Mrs. Hewer issued her well-known handbook on the same subject, to bring out yet another book. The worst of it is that we are threatened with a series of manuals on nursing, of which this of Mr. Bishop's is the first, and every one knows that the market is already flooded with cheap books on nursing, and that what is really wanted is the one transcendent all-comprehensive work, founded on these fugitive efforts of many people. One number of the series we shall look for with interest, and if really well done it should fill a want in the nursing world; namely, "A complete Glossary and Dictionary of terms used in Nursing." But we wish we could have been spared more handbooks on the medical, surgical, and obstetric part of the subject.

### Food in Motherhood.†

This is a thoroughly American book, full of the quaint anecdotes and short, sharp sayings which our cousins across the pond excel in; and probably because written in this familiar and amusing style, the book will do much good. Far be it from us though to become such believers in beef as Ephraim Cutter! Let us grant there is a little nourishment in mutton, and lighter diet generally, and let us uphold the reputation of Scotch porridge. Over here oatmeal is not "sold at a high price," and beef is; surely then it must be the force of circumstance which makes an American declare it is possible only to flourish on beef, and a Scotchman to attribute his good health to oatmeal. Dr. Cutter says: "Oats are good for horses and bovines. Man had better leave oats to the horses;" but that is because poor Dr. Cutter knows no better. Again: "Beef is the only article of solid animal food on which man can live and thrive for any length of time indefinitely;" most evidently Dr. Cutter has never made the acquaintance of a Highlander reared on oatmeal, tasting beef never, and mutton only at infrequent intervals. But leaving out the question whether Americans do not owe much ill-health to the cheapness of meat, this book is a good one, because it forcibly brings the doctrine of the supreme importance of diet, both to mother and child, well to the fore. All alcohol is strictly forbidden—another American trait—and many an English mother would stare aghast if the time-honoured glass of stout were refused her. The book reminds us of the English monthly nurse who attended a Hindoo lady, and who was astonished at the frequent baths, strange food, and queer customs which were gone through, in spite of all protests on her part. "But," the nurse naively concluded, "though the mother broke every obstetrical rule taught in England, both she and the child are doing well."

* "Lectures to Nurses on Antiseptics in Surgery." By E. Stanmore Bishop, F.R.C.S. (Sampson Low). Price 2s.

† "Food in Motherhood." By Ephraim Cutter. M.D. Harv. (David Stott.) Price 1s.

### Everybody's Opinion.

*[Correspondence on all subjects is invited, but we cannot in any way be responsible for the opinions expressed by our correspondents. No communications can be entertained if the name and address of the correspondent is not given, or unless one side of the paper only be written on.]*

### A HOLIDAY HOME.

MISS F. M. HUGHES writes:—Will you allow me to say a few words respecting Sharnhen House, Folkestone, a home of rest for nurses and professional gentlewomen? I spent a few days at the home recently, and can speak most highly of the comfortable and prettily-furnished rooms, the excellently cooked and abundant fare, and last, but not least, the kindly and courteous welcome of the ladies in charge. The terms are very moderate, and are inclusive. I would recommend all nurses who have no home at which to spend their holidays to try this one, and I feel sure that they will not regret their choice.

### METROPOLITAN AND NATIONAL DISTRICT NURSES.

FIDELE writes: Having some time ago been trained at 23, Bloomsbury Square, as a district nurse, I went on Friday last to the annual meeting of the Central Home, held at Grosvenor House, hoping to hear how my old friends in Bloomsbury Square were getting on. It was a terribly wet day; many of the speakers failed, and the whole affair was generally very limp. But what struck me, and many friends who have spoken to me about it since, was that little or no information was given about the work now doing in the Home, and though the speakers congratulated one another greatly on the high standard attained by the nurses of the in-titution. When I was in the Home we were all enthusiastic admirers of our Superintendent, Miss Mansel, and I am sure all Bloomsbury nurses feel how much of their power to alleviate suffering they owe to her excellent skill in teaching the difficult duties of district nursing; but of her not a word was said at the meeting. This mistake at the meeting left a very confused impression on the minds of many, some thinking that the Central Home had moved to Paddington, others fearing that Miss Mansel had left the work altogether, so that it has given rise to many vague surmises. I find, however, after making due inquiries, that the work of training ladies as nurses for the poor is still carried on in Bloomsbury Square, and if any of your readers are thinking of joining that good work, I can assure them they will never regret taking that step, for I always look back upon the few months I spent in the Central Home as among the happiest of my life.

### JUNIUS S. MORGAN MEMORIAL.

NURSE ADA writes: I have more than £5 collected, but it is possible others may have less than that sum, as I am glad you say we are each to do what we can. I am going to knit my purse, so I hope the Princess may put it to some use. Let us make a holiday of the presentation day, and not fret about uniforms being all alike; if we are neat and tidy, and each in our own uniform, surely that will be all that is needed.

### Presentations.

BARNHILL HOSPITAL, GLASGOW.—Miss Blumenthal, who has been appointed matron of the Fulham Union Infirmary, was presented on May 8th with a beautiful dressing-bag by the nurses of this institution. The presentation was made by Dr. Core, medical superintendent, who referred to the pleasing relations which had existed between Miss Blumenthal and everyone connected with the management during the four years she had filled the post of lady superintendent.

LYING-IN HOSPITAL.—A pleasing incident took place at this hospital, City-road, London, on May 13, when the Night Superintendent (Miss Ormerod) was presented with a railway rug and straps, by some of the nurses and midwives, as a token of their appreciation. Miss Ormerod was trained in the Nightingale School for Nurses, at St. Thomas's Hospital; from there she went to the Marylebone Infirmary, where she remained three and a-half years; she then took the post of night superintendent in the City Road Lying-in Hospital; she is soon leaving to take up the matronship of the Lying-in Hospital in Belfast.

ROYAL HOSPITAL, PORTSMOUTH.—Miss Alice Giles, who has lately resigned her post of head nurse of the women's floor of this hospital, has been presented by the nurses and probationers with a handsome travelling clock. Also, at the same institution, Miss Annie Ellis, on her leaving, was presented with a handsome photographic album. She had the post of head nurse of the men's ward. Both received excellent testimonials from the medical staff.

### Appointments.

Bristol General Hospital.—Miss Eaton has been appointed night superintendent at the Bristol General Hospital. Miss Eaton was trained at the Derbyshire General Infirmary, where she afterwards held the post of head nurse for five years. Last November Miss Eaton was appointed sister of the Norman and Adams' wards at the Royal Albert Hospital, Devonport, and she takes with her the good wishes of every-one to her new duties at Bristol.

Boscombe Hospital.—On April 30th, Miss F. M. Tomassen was appointed matron of this hospital, near Bournemouth. She trained at Preston, and then joined the Sheffield Institute; later, Miss Tomassen took charge of a male surgical ward at Bristol Infirmary. Lately Miss Tomassen has been head nurse to a surgical home in Fitzroy Square, where she nursed some cases for Sir Joseph Lister, who gives her a good testimonial. Boscombe is a delightful little hospital, and we are sure the new matron will be happy there.

St. Mary Abbott's Infirmary.—Miss Adeline Griffiths has been appointed assistant matron of this infirmary at Kensington. Miss Griffiths, trained at Huddersfield, worked at the Seamen's Hospital from 1882 to 1884, went to Sydney, and had charge of a pay hospital; and for the last eighteen months has been charge nurse at the Hospital for Ladies, 90, Harley Street. Miss Griffiths possesses excellent certificates from some of the most competent doctors and matrons in the land, and we are sure that St. Mary Abbott's Infirmary is to be congratulated on having obtained such an assistant to its matron.

West Cornwall Infirmary.—Miss A. E. Whittaker has been appointed matron of this important infirmary at Penzance. Miss Whittaker trained at Bradford Nurses' Institution, which she left in 1880 to go to Bradford Infirmary. Miss Whittaker subsequently served for a short time at the Clayton Hospital, Wakefield, and at Essex and Colchester Hospitals. Lately she has been employed as matron at the G.W.R. Accident Hospital at New Swindon, where for six years she has worked well and won golden opinions both as a nurse and a manager.

### The National Pension Fund for Nurses.

Many noblemen and gentlemen are becoming governors of this Fund. Lord Derby, writing to the founder of the Fund on the 17th inst., says: "I have too many other claims upon me to help your Nurses' Pension Fund substantially, but as a mark of goodwill I enclose a cheque for £25 as a donation." Lord Derby did not give until he had fully considered the matter and gone carefully into the accounts, and we have no doubt his action will be of material assistance to the Fund in many ways, as he is universally recognised as being a man of sound judgment combined with the highest intelligence and common sense.

### Notes and Queries.

### Queries.

(14) *Japan.*—Can anyone tell me whether English nurses are wanted in Japan, and how to set about getting work there?—*Nightingale Nurse.*

### Answers.

*Nurse Mildred.*—Sister Rose Gertrude's address is Kalihi Oaha, Hawaian Islands, but more nurses are not wanted there, nor could you go at Government expense.

*A. S.*—We hope to secure you an invitation to be present and hand in your purse.

*E. J.*—We are doing what we can, but the line must be drawn somewhere.

*Nurses' Co-operation.*—All inquirers on this subject must please wait patiently. The provisional committee are now settling details, and the scheme will be published in this paper when concluded.

*E. A. J.*—You can demand a copy of the agreement, and if they do not give it you it will be to their discredit. But you cannot force it from them unless they take legal proceedings against you. You are bound by the agreement, and if it says nothing and implies nothing about going abroad, or to other institutions, you can refuse.

### Vacancies.

The following vacancies are announced:

### Matrons.

Colonial Hospital, Gibraltar; £70.
Kendal Hospital; £45.

### Sister.

Albert Hospital, Devonport.
Monsall Fever Hospital; £32.

### Nurses.

Aberdeen Poorhouse; £22.

Alexandra Hospital for Children, Brighton; £20.

Arbroath Poorhouse; £30.

Birkdale Nurses' Home.

Birmingham Hospital for Women; £23.

Blackheath Institute.

Bristol Royal Infirmary (superintendent); £40.

Burton-on-Trent Institution; £25.

Cancer Hospital, Brompton; £25.

Canterbury Nurses' Institute.

Carmarthen Infirmary; £23.

Cheltenham Hospital.

East London Nursing Institute.

Frome Nurses' Home; £22.

Fulham Union; £18.

Fylde Union; £20.

General Nursing Institute.

Guildford Isolation Hospital.

Hanover Institute for Nurses; £25.

Harrogate Bath Hospital; £25.

Hollond Institution, Nice.

Huddersfield Nurses' Home.

Isle of Wight Infirmary; £18.

Jersey Institution; £25.

Kendal Hospital; £28.

Kidderminster Infirmary; £25.

Kent and Canterbury Hospital (night); £24.

Kingston Hill Convalescent Home; £20.

Leeds Nurses' Institute; £25.

Levick Institution, Paris.

Lewisham Union; £30.

Manchester and Salford Nursing Institution; £25.

Mrs. Mann's Surgical Home; £24.

Newcastle Infirmary; £20.

Newcastle City Hospital; £30.

Nurses' Institute, Weston-super-Mare.

Nurses' Institute, Surbiton.

Pembrokeshire Infirmary, Haverfordwest.

Pendlebury Hospital for Children; £20.

Shoreditch Infirmary; £20.

St. Helena Home, N.W.

Stoke-on-Trent Nurses' Home.

St. Pancras Workhouse; £20.

St. Peter's Nurses' Institution, Bedford; £27.

St. Saviour's Infirmary; £20.

St. Vanda's Home, Bromley.

West Bromwich District Hospital; £25.

### District Nurses.

Canterbury Nurses' Institute.

Camborne, Cornwall.

East London Nursing Society.

Hulme (Manchester) District Nursing Institution; £25.

Queen Victoria's Nursing Institute (Scotch Branch).

Seal, Sevenoaks.

Stoke-on-Trent Nurses' Home.

Worcester City Institution.

### Probationers.

Bobbacombe Hospital for Children.

Bradford Fever Hospital; £12.

Buxton Devonshire Hospital.

Highgate Hospital for Children.

Monsall Fever Hospital; £15.

Newcastle Nurses' Home.

North Cambridge Hospital.

Pembrokeshire Infirmary, Haverfordwest.

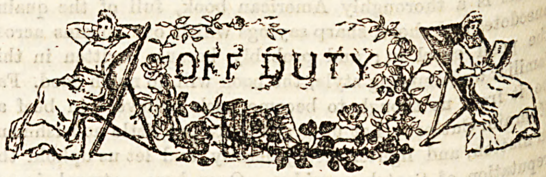

### Amusements and Relaxation.

**N.B.—New Word Competition Commenced April 5th, 1890, ends June 28th, 1890.**

Proper names, abbreviations, foreign words, words of less than four letters, and repetitions are barred; plurals, and past and present participles of verbs, are allowed. Nuttall's *Standard* dictionary only to be used.

The word for dissection for this, the EIGHTH week of the quarter, being

"LABURNUMS."

| Names. | May 15th. | Totals. | Names. | May 15th. | Totals. |
|---|---|---|---|---|---|
| Adeline | — | 149 | Agamemnon | 34 | 218 |
| Patience | 36 | 224 | G. E. J. C. | — | 34 |
| Lightowlers | 28 | 208 | S. E. A. | — | 24 |
| Canary | — | 86 | Jenny Wren | 33 | 210 |
| M. E. S. | — | 37 | Multum in parvo | — | 32 |
| Ambition | — | 36 | Ecila | — | 39 |
| M. W. | 35 | 217 | Weta | 35 | 179 |
| Esperance | 35 | 218 | Henri | — | 161 |
| Judy | — | 37 | Holland | 36 | 220 |
| Reldas | 36 | 222 | T. J. | — | 111 |
| Merenda | — | 29 | Nurse Isabel | — | 17 |
| Lucy Locket | — | 40 | Nurse Faith | — | 9 |
| Camellia | — | 30 | E. S. Z. | — | 10 |
| Qu'appelle | 36 | 217 | G. P. | — | 10 |
| Tinie | 35 | 224 | Stumps | — | 33 |
| Nurse Mildred | 26 | 163 | Bolton | — | 8 |
| Coralie | 35 | 220 | Jesmur | — | 53 |
| Gladys | 35 | 220 | Ran | 17 | 17 |

### Notice to Correspondents.

N.B.—Each paper must be signed by the author with his or her real name and address. A *nom de plume* may be added if the writer does not desire to be referred to by us by his real name. In the case of all prize-winners, however, the real name and address will be published.

Competitors can enter for *all* quarterly competitions, but no competitor may take more than *one first prize* during the year.

